# Dr. Henry Parke Custis Wilson

**Published:** 1898-02

**Authors:** 


Dr. Henry Parke Custis Wilson, of Baltimore, died at his home in that city Monday, December 27, 1897, aged 70 years. He took the degree of A. B. at Princeton in 1848, and his doctorate degree from the University of Maryland in 1851. He was descended from the Custis family, hence was a relative of Martha Washington. He was one of the founders of the American gynecological society and served as its president in 1889. He was also a member of local, state and national medical bodies, as well as an honorary member of many foreign societies. He enjoyed the personal friendship of the most prominent physicians at home and abroad and was easily one of the most distinguished physicians of his time. Dr. Wilson lost a son by drowning nearly two years ago, which was a sad blow in his declining years. He leaves a widow and five children, one of whom is Dr. Robert T. Wilson, of Baltimore.

